# Perlecan, A Multi-Functional, Cell-Instructive, Matrix-Stabilizing Proteoglycan With Roles in Tissue Development Has Relevance to Connective Tissue Repair and Regeneration

**DOI:** 10.3389/fcell.2022.856261

**Published:** 2022-04-01

**Authors:** Anthony J. Hayes, Brooke L. Farrugia, Ifechukwude J. Biose, Gregory J. Bix, James Melrose

**Affiliations:** ^1^ Bioimaging Research Hub, Cardiff School of Biosciences, Cardiff University, Wales, United Kingdom; ^2^ Department of Biomedical Engineering, Melbourne School of Engineering, The University of Melbourne, Melbourne, VIC, Australia; ^3^ Departments of Neurosurgery and Neurology, Clinical Neuroscience Research Center, Tulane University School of Medicine, New Orleans, LA, United States; ^4^ Graduate School of Biomedical Engineering, University of New South Wales, Sydney, NSW, Australia; ^5^ Raymond Purves Bone and Joint Research Laboratories, Kolling Institute of Medical Research, Royal North Shore Hospital, The Faculty of Medicine and Health, The University of Sydney, St. Leonard’s, NSW, Australia

**Keywords:** perlecan, repair biology, vascular repair, cartilage repair, repair of blood brain barrier, perlecan domain-I, perlecan domain-V, growth factor delivery

## Abstract

This review highlights the multifunctional properties of perlecan (HSPG2) and its potential roles in repair biology. Perlecan is ubiquitous, occurring in vascular, cartilaginous, adipose, lymphoreticular, bone and bone marrow stroma and in neural tissues. Perlecan has roles in angiogenesis, tissue development and extracellular matrix stabilization in mature weight bearing and tensional tissues. Perlecan contributes to mechanosensory properties in cartilage through pericellular interactions with fibrillin-1, type IV, V, VI and XI collagen and elastin. Perlecan domain I - FGF, PDGF, VEGF and BMP interactions promote embryonic cellular proliferation, differentiation, and tissue development. Perlecan domain II, an LDLR-like domain interacts with lipids, Wnt and Hedgehog morphogens. Perlecan domain III binds FGF-7 and 18 and has roles in the secretion of perlecan. Perlecan domain IV, an immunoglobulin repeat domain, has cell attachment and matrix stabilizing properties. Perlecan domain V promotes tissue repair through interactions with VEGF, VEGF-R2 and α2β1 integrin. Perlecan domain-V LG1-LG2 and LG3 fragments antagonize these interactions. Perlecan domain V promotes reconstitution of the blood brain barrier damaged by ischemic stroke and is neurogenic and neuroprotective. Perlecan-VEGF-VEGFR2, perlecan-FGF-2 and perlecan-PDGF interactions promote angiogenesis and wound healing. Perlecan domain I, III and V interactions with platelet factor-4 and megakaryocyte and platelet inhibitory receptor promote adhesion of cells to implants and scaffolds in vascular repair. Perlecan localizes acetylcholinesterase in the neuromuscular junction and is of functional significance in neuromuscular control. Perlecan mutation leads to Schwartz-Jampel Syndrome, functional impairment of the biomechanical properties of the intervertebral disc, variable levels of chondroplasia and myotonia. A greater understanding of the functional working of the neuromuscular junction may be insightful in therapeutic approaches in the treatment of neuromuscular disorders. Tissue engineering of salivary glands has been undertaken using bioactive peptides (TWSKV) derived from perlecan domain IV. Perlecan TWSKV peptide induces differentiation of salivary gland cells into self-assembling acini-like structures that express salivary gland biomarkers and secrete α-amylase. Perlecan also promotes chondroprogenitor stem cell maturation and development of pluripotent migratory stem cell lineages, which participate in diarthrodial joint formation, and early cartilage development. Recent studies have also shown that perlecan is prominently expressed during repair of adult human articular cartilage. Perlecan also has roles in endochondral ossification and bone development. Perlecan domain I hydrogels been used in tissue engineering to establish heparin binding growth factor gradients that promote cell migration and cartilage repair. Perlecan domain I collagen I fibril scaffolds have also been used as an FGF-2 delivery system for tissue repair. With the availability of recombinant perlecan domains, the development of other tissue repair strategies should emerge in the near future. Perlecan co-localization with vascular elastin in the intima, acts as a blood shear-flow endothelial sensor that regulates blood volume and pressure and has a similar role to perlecan in canalicular fluid, regulating bone development and remodeling. This complements perlecan’s roles in growth plate cartilage and in endochondral ossification to form the appendicular and axial skeleton. Perlecan is thus a ubiquitous, multifunctional, and pleomorphic molecule of considerable biological importance. A greater understanding of its diverse biological roles and functional repertoires during tissue development, growth and disease will yield valuable insights into how this impressive proteoglycan could be utilized successfully in repair biology.

## Introduction

**FIGURE 1:**
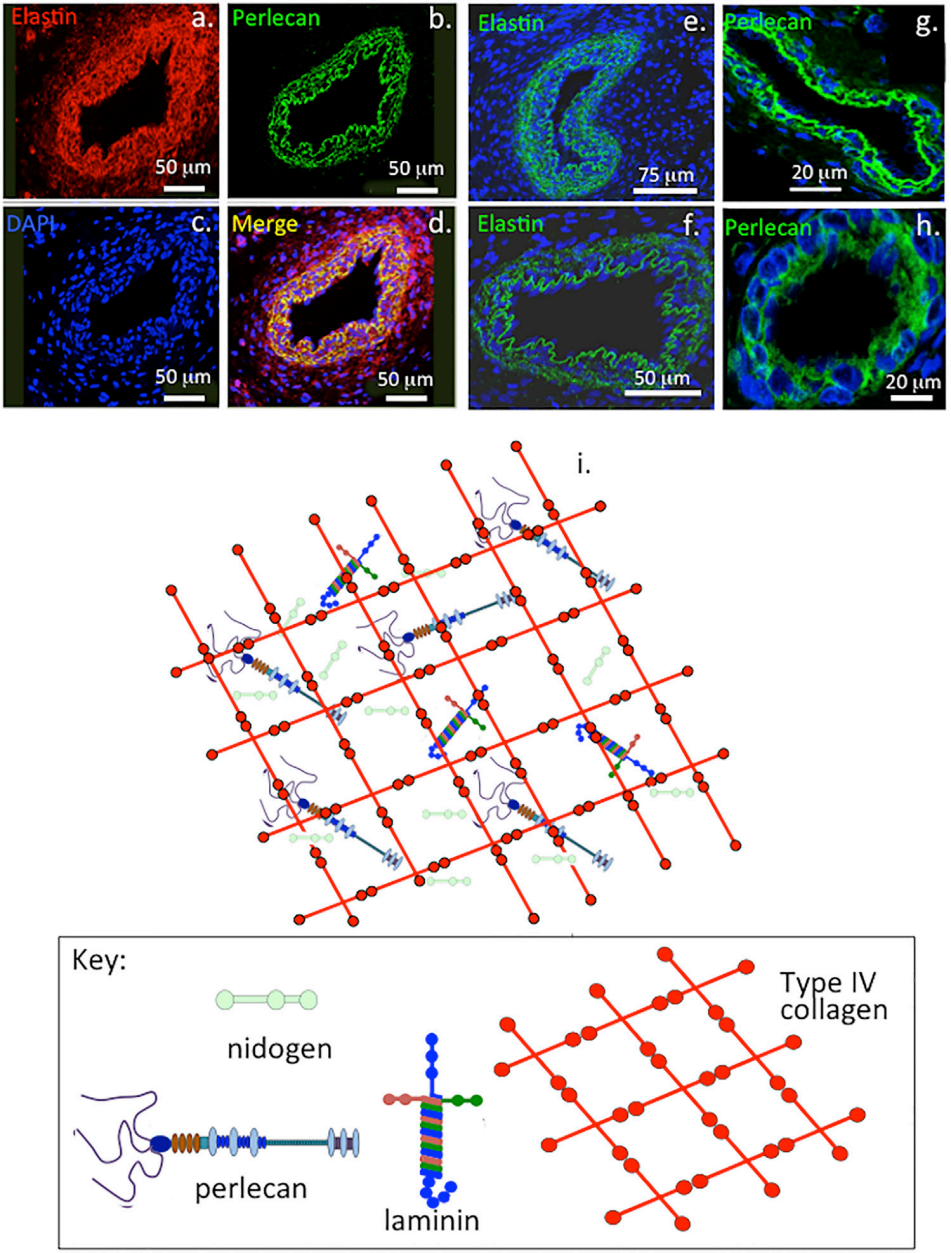
Immunofluorescent colocalisation of perlecan and elastin in transverse sections of blood vessels **(A–D)** and immunolocalisation of elastin **(E,F)** and perlecan **(G,H)** in venules **(E,G)** and capillaries **(F,H)**. The underlying schematic depicts the major extracellular matrix components of basement membranes highlighting perlecan’s interactive role in maintaining integrity of this structure **(I)**. **(A–H)** reproduced from (Hayes et al., 2011a) with permission.

This review highlights the interactive properties of perlecan (HSPG2) and its potential applications in repair biology. Perlecan is often referred to as a large heparan sulfate proteoglycan (HS-PG) but exists as a HS/chondroitin sulfate (CS) hybrid form in most tissues, endothelial cells however synthesize a monosubstituted HS glycoform (Melrose et al., 2008). Keratinocytes produce a form of perlecan substituted with keratan sulfate (KS), HS and CS side chains and it is one of only a few PGs, which are found with such a glycosaminoglycan (GAG) substitution pattern (Knox et al., 2005). Perlecan, domain I contains three 70–100 kDa HS or CS chains attached to serine 65, 71, and 76, mouse perlecan also contain a HS or CS substitution site on Ser 3593 or Serine 3250 respectively on domain V (Tapanadechopone et al., 1999). Perlecan has a ubiquitous distribution and occurs in vascular, poorly vascularized cartilaginous, fibro-cartilaginous, adipose, lymphoreticular systems, neural, bone and bone marrow stromal tissues (Melrose et al., 2008). Perlecan is a major basement membrane component (Figure 1) as are type IV collagen, laminin and nidogen. PRELP (proline/arginine-rich end leucine-rich repeat protein), a HS binding SLRP (small leucine rich proteoglycan) and type IV collagen both interact and anchor perlecan in the vascular basement membrane (Bengtsson et al., 2002). The basement membrane is a widely distributed, specialized, thin, dense, sheet-like structure tailored to the needs of specific tissues and organs with roles as a cellular scaffold and cell signaling platform (Timpl, 1996; Pozzi et al., 2017). While cartilaginous tissues are devoid of sheet-like basement membrane structures it has been suggested that the pericellular matrix (PCM) surrounding each chondrocyte serves a similar role (Kvist et al., 2008). Von Willebrand factor A‐domain‐related protein (WARP) also interacts with perlecan, as a bridging structure to type VI collagen in the chondrocyte PCM (Allen et al., 2006; Fitzgerald, 2020), and stabilizes the basement membrane of basal structures in peripheral nerves (Allen et al., 2008; Allen et al., 2009). WARP is found in a distinct sub-set of nerve basement membrane and neural vasculature (Allen et al., 2008; Fitzgerald, 2020).

**FIGURE 2:**
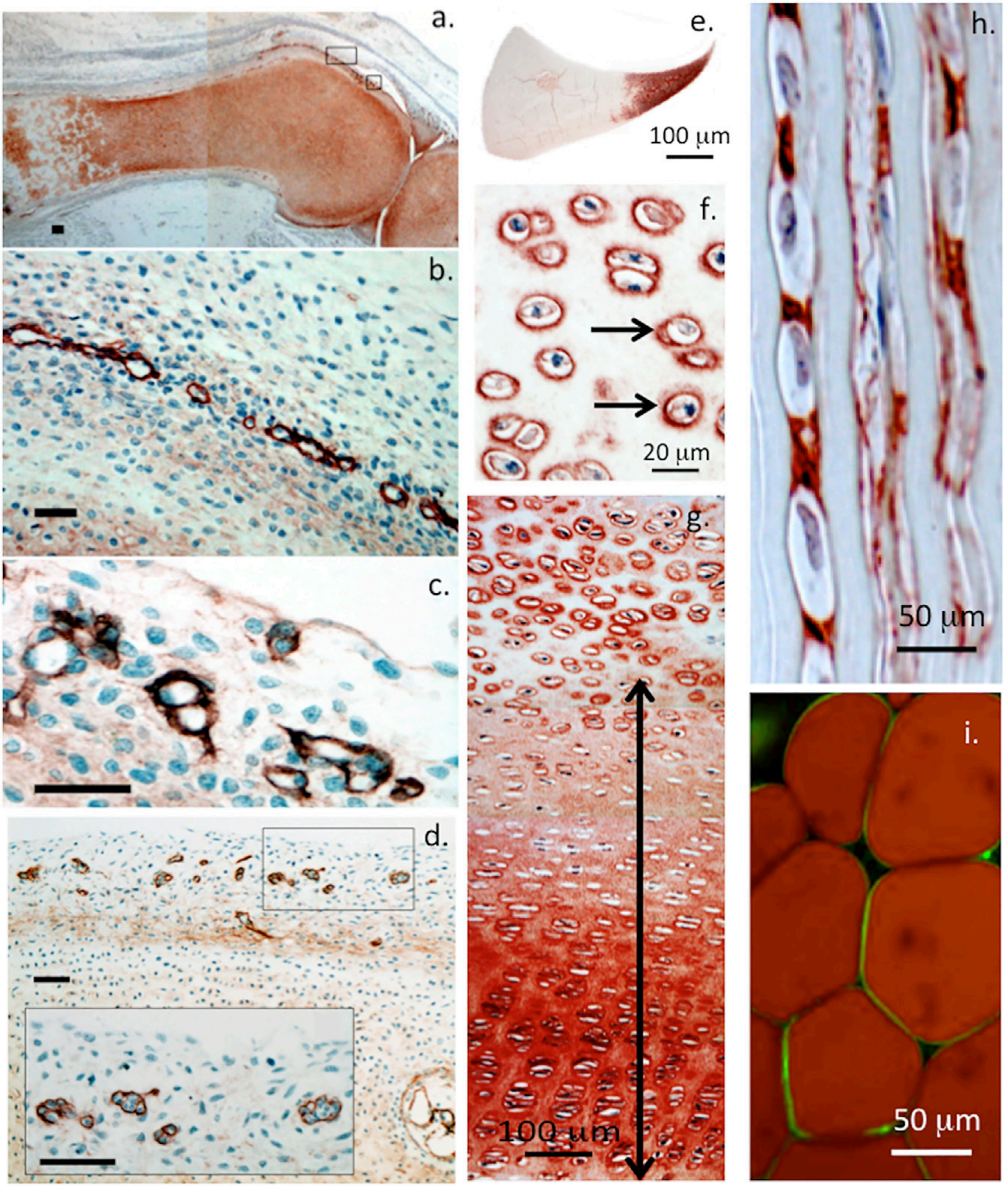
Immunolocalisation of perlecan in 14-week-old human foetal knee joint **(A–D)**, newborn ovine cartilaginous tissues **(E–H)** and mature human adipocytes **(I)**. Perlecan has a diffuse distribution in cartilage rudiments in the femur and tibia of a foetal knee joint **(A)**. The boxed areas in **(A)** are depicted at higher magnification in **(B,C)**. These show strong localization of perlecan in putative stem cell niches in the surface region of the cartilage rudiments **(B,C)** and diffuse extracellular staining in the rudiment. Perlecan localization in putative stem cell niches within the surface regions of a hip rudiment **(D)**. The boxed area in **(D)** is depicted at higher magnification in the inset. Scale bars in **(A–D)**, 50 μm. Macroscopic immunolocalisation of perlecan in a newborn ovine medial meniscus. Perlecan is concentrated predominantly within the inner cartilage-like meniscal zone **(E)**. Pericellular immunolocalisation (arrows) of perlecan within neonatal ovine femoral head articular cartilage **(F)** and resting zone tibial growth plate chondrocytes. Double headed arrow indicates extracellular gradient of perlecan immunolabel extending from the resting zone through to the columnar proliferating and hypertrophic growth plate chondrocytes **(G)**. Polarised pericellular immunolocalisation of perlecan in strings of cells in the newborn ovine ACL **(H)**. Pericellular immunolocalization of perlecan around human adipocytes **(I)**. **(A–D)** modified from (Melrose and Melrose, 2016) with permission, images © Melrose 2016, **(E–H)** modified from (Smith et al., 2010), **(I)** reproduced from (Yamashita et al., 2018) with permission.

Perlecan colocalizes with elastin in blood vessels and this contributes to their visco-elastic properties in vasculogenesis during establishment of early vascular networks and tissue development (Hayes et al., 2011a) and is also a key component of basement membranes (Figure 1). Perlecan occurs in specialized neuroprogenitor stem cell niches termed fractones in the sub-ventricular and sub-granular dentate gyrus of the hippocampus where it promotes neural cell survival and proliferation through sequestered FGF-2 (Kerever et al., 2014; Kerever et al., 2007). Perlecan is also associated with chondroprogenitor cell niches in cartilage rudiments where it promotes the attainment of stem cell pluripotency and migratory stem cell lineages that participate in joint cavitation, cartilage development and the expansion of cartilaginous rudiments (Hayes et al., 2016a; Melrose and Melrose, 2016) (Figure 2). Perlecan promotes chondrocyte proliferation and differentiation and ECM production and the assembly of a transient developmental cartilaginous scaffold (Melrose et al., 2016). Perlecan is up-regulated in hypertrophic chondrocytes that establish the primary and secondary ossification centers in the rudiments, these will give rise to the long bone cartilage growth plates (Melrose et al., 2004; Smith et al., 2010). Perlecan also promotes the establishment of primitive vascular networks in the stromal tissues surrounding fetal rudiments that provide nutrition to the rapidly expanding cell numbers within the mesenchymal condensations and cartilage rudiments (Melrose et al., 2003; Shu et al., 2013). Perlecan provides mechanical stability to the developing ECM through interactions with a range of HS-binding structural matrix components. Pericellular type VI and XI collagen interactions with perlecan aids in the stabilization and functional properties of mature cartilaginous ECMs (Hayes et al., 2016b; Smith and Melrose, 2019; Guilak et al., 2021). Interactions of perlecan with a wide range of HS interactive structural matrix proteins and cell adhesive glycoproteins, ensure efficient cell-matrix communication with weight bearing and tensional stresses providing mechanotransductive signals to chondrocytes to maintain tissue homeostasis (Knox and Whitelock, 2006; Melrose et al., 2008; Melrose, 2020). Perlecan contributes to mechano-sensory regulatory properties in cartilaginous tissues mediated by pericellular interactions with type VI and XI collagen, fibrillin-1 and elastin (Hayes et al., 2013; Hayes et al., 2016b; Smith and Melrose, 2019; Melrose, 2020; Guilak et al., 2021; Hayes et al., 2021).

**FIGURE 3:**
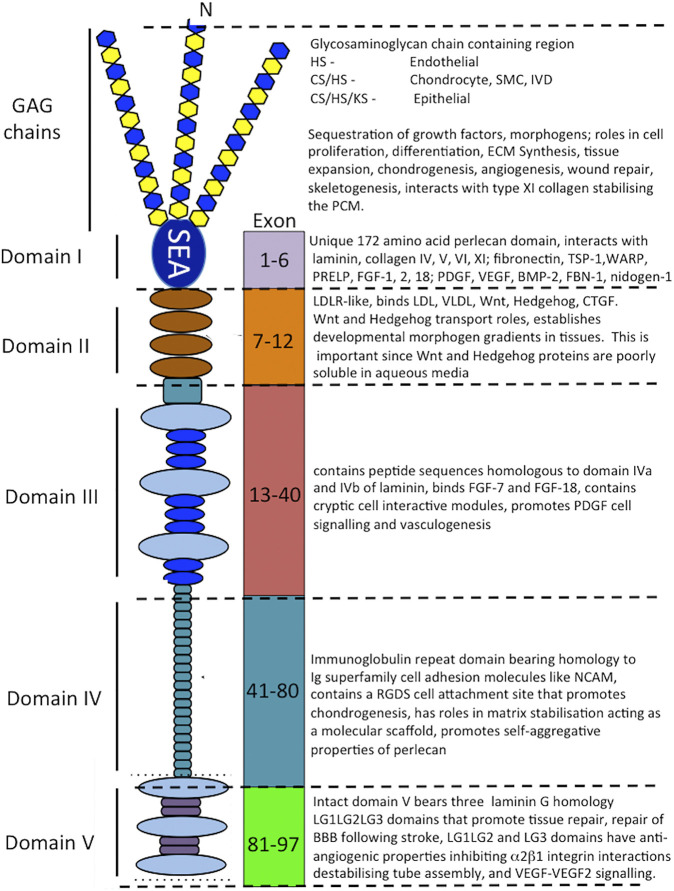
Schematic of the modular structure of perlecan and the interactive and cell instructive properties of each of its domains.

Perlecan is a modular multifunctional PG with five distinct domains. Domain I is unique to perlecan and its GAG chains bind fibroblast growth factors (FGFs), platelet-derived growth factor (PDGF), vascular endothelial growth factor (VEGF) and bone morphogenetic proteins (BMPs) promoting cellular proliferation, differentiation and tissue development (Melrose et al., 2008; Knox and Whitelock, 2006; Melrose, 2020; Whitelock et al., 2008) (Figure 3). Perlecan-HS mediated interactions with ECM components stabilize tissues (Arikawa-Hirasawa et al., 1999; Costell et al., 1999; Nicole et al., 2000; Gomes et al., 2002; Mongiat et al., 2003; Knox and Whitelock, 2006; Melrose et al., 2006; Farach-Carson and Carson, 2007; Rodgers et al., 2007; Kvist et al., 2008; Melrose et al., 2008; Smith et al., 2010; Hayes et al., 2011a; Hayes et al., 2011b; Singhal and Martin, 2011; Thompson et al., 2011; Hayes et al., 2013; Shu et al., 2013; Hayes et al., 2014; Wilusz et al., 2014; Shu et al., 2016a; Hayes et al., 2016b; Sadatsuki et al., 2017; Smith and Melrose, 2019; Melrose, 2020; Ocken et al., 2020). Domain II bears homology with low-density lipoprotein (LDL) receptor and has roles in the clearance of LDL and very low-density lipoprotein (VLDL) from the bloodstream. Domain II binds the poorly soluble Wnt and Hedgehog morphogens and acts as a transport PG, establishing gradients of these morphogens in tissues. Domain III of perlecan binds FGF-7 and 18 (Mongiat et al., 2000; Smith et al., 2007) and domain IV, an immunoglobulin (Ig) repeat domain has cell attachment properties, bears homology with the cell membrane Ig receptor family and neural cell adhesion molecule (NCAM) and also has roles as a scaffolding material (Noonan et al., 1991; Murdoch et al., 1992; Hopf et al., 1999; Farach-Carson et al., 2008; Martinez et al., 2018).

The carboxyl terminal domain V of perlecan contains three laminin-type G domains (LG) and four EGF-like repeats (Noonan et al., 1991; Murdoch et al., 1992). These LG domains are homologous with the α chain globular domains of laminin and facilitate cell-ECM interactions (Timpl, 1996) and unique divergent roles in angiogenesis, vascular cell interactions, wound healing and autophagy (Gubbiotti et al., 2017). Missense mutations, alternatively spliced, truncated or ablated sequences in perlecan domain V are evident in an autosomal recessive disorder called Schwartz Jampel Syndrome (SJS) characterized by neuromuscular deficits and myotonia (Gubbiotti et al., 2017). Domain V interacts with α2β1 integrin on endothelial cells in the assembly of angiogenic capillary tubes (Lord and Whitelock, 2013). Recombinant perlecan domain V has been expressed using human, mouse and *Drosophila* (domain V homologue unc-52) DNA sequences in bacterial and mammalian expression systems (Lord and Whitelock, 2013). Human domain V sequence from Glu3687 to Ser4391 when expressed in HEK-293 cells resulted in the synthesis of a GAG-free perlecan domain V, this was termed endorepellin. However when mouse perlecan domain V (Brown et al., 1997; Friedrich et al., 1999; Tapanadechopone et al., 1999), or human domain V encompassing 37 amino acids of the C-terminal region of perlecan domain IV from Leu3626 to Ser439 was expressed in HEK-293 cells a perlecan domain V containing HS and CS chains was produced. _rh_Perlecan domain V is a fully functional vascular PG in its own right supporting endothelial cell interactions as effectively as full-length perlecan thus it has considerable promise in repair biology (Lin et al., 2020).

Endogenously produced perlecan domain V released by MMPs from full-length perlecan also promotes tissue repair through angiogenic interactions with VEGF, VEGF-R2, α2β1 integrin, ECM-1 and progranulin (Brown et al., 1997; Bix, 2013; Bix et al., 2013). LG1-LG2 and LG3 modules of perlecan domain V antagonize these interactions and inhibit tube formation and in-growth of new blood vessels. Perlecan domain V promotes repair of the disrupted blood brain barrier that occurs after ischemic stroke (Bix, 2013; Bix et al., 2013). Interactions between perlecan domain V and pro-angiogenic glycoproteins such as extracellular matrix protein 1 (ECM1) (Mongiat et al., 2003) and progranulin (Gonzalez et al., 2003) promote angiogenesis and tissue repair. Perlecan complexes with dystroglycan and acetylcholinesterase occur in the neuromuscular junction (NMJ) and are of functional significance with essential roles in the assembly and function of this structure. Perlecan domain V α5β1 integrin interactions promote pericyte migration, enhance PDGF-BB-induced phosphorylation of platelet-derived growth factor receptor β (PDGFRβ), SHP-2 (Src homology region 2 domain-containing phosphatase-2), and focal adhesion kinase (FAK) (Nakamura et al., 2019). This supports the maintenance of the normal blood brain barrier and repair processes of this structure following ischemic stroke (Nakamura et al., 2015a; Nakamura et al., 2019). Perlecan domain V and its LG1LG2 and LG3 modules differentially modulate interactions with VEGF-1, 2; FGF-7; PDGF promoting or inhibiting angiogenesis. Perlecan domain V is neuroprotective, and has anti-inflammatory properties leading to the proposal of domain-V for the treatment of Alzheimer’s disease (AD), stroke patients (Lee et al., 2011; Clarke et al., 2012; Kahle et al., 2012; Bix, 2013; Bix et al., 2013; Guell and Bix, 2014; Marcelo and Bix, 2014; Edwards and Bix, 2019a; Edwards and Bix, 2019b), vascular dementia (Marcelo and Bix, 2015) and brain trauma (Badaut and Bix, 2014). Perlecan and VEGF incorporated into bio-scaffolds promote angiogenesis and tissue repair (Rnjak-Kovacina et al., 2016; Lord et al., 2017). Recombinant perlecan domain V supports angiogenesis, vascular cell interactions and wound healing (Lee et al., 2011; Clarke et al., 2012; Kahle et al., 2012; Marcelo and Bix, 2014; Marcelo and Bix, 2015; Poluzzi et al., 2016; Rnjak-Kovacina et al., 2017). Interactions between perlecan PDGF BB, FGF-2, and TGF-β1 promote fibroblast migration and collagenous repair of the corneal stroma and other connective tissues to regulate tissue fibrosis. Perlecan-VEGF-VEGF-R2 interactions promote angiogenesis and wound healing (Lord et al., 2014). Perlecan domain III and V interactions with platelet factor 4 and domain I of perlecan with megakaryocyte and platelet inhibitory receptor G6b-R promote adhesion of cells to implants and scaffolds in vascular repair applications (Rnjak-Kovacina et al., 2017). With the availability of recombinant perlecan, applications are now emerging for perlecan in repair biology. Perlecan expression by adipocytes and smooth muscle cells mechano-regulate lipid and glucose catabolism and oxidative muscle fiber composition and the systemic metabolism of adipose tissue and skeletal muscle (Yamashita et al., 2018). Perlecan thus has physiological roles in obesity, the onset of metabolic syndrome and in lipoprotein retention in diabetic atherosclerosis (Tannock and King, 2008; Ng et al., 2021). Polarised M2 macrophages retain LDL in atherosclerotic plaques through interactions with perlecan domain I HS chains and the LDL receptor of perlecan domain II. Perlecan as a basement membrane component of blood vessels, blood brain barrier and nerve basal structures in moto-neuron synapses of the NMJ is thus a ubiquitous pleomorphic molecule involved in the regulation of many physiological processes in tissues of varied form and function. With a full appreciation of perlecan’s biology in tissues this insightful information would greatly aid in future tissue repair strategies involving this remarkable PG.

## The Diverse Roles of Perlecan

### Perlecan’s Roles in Tissue Development

**FIGURE 4:**
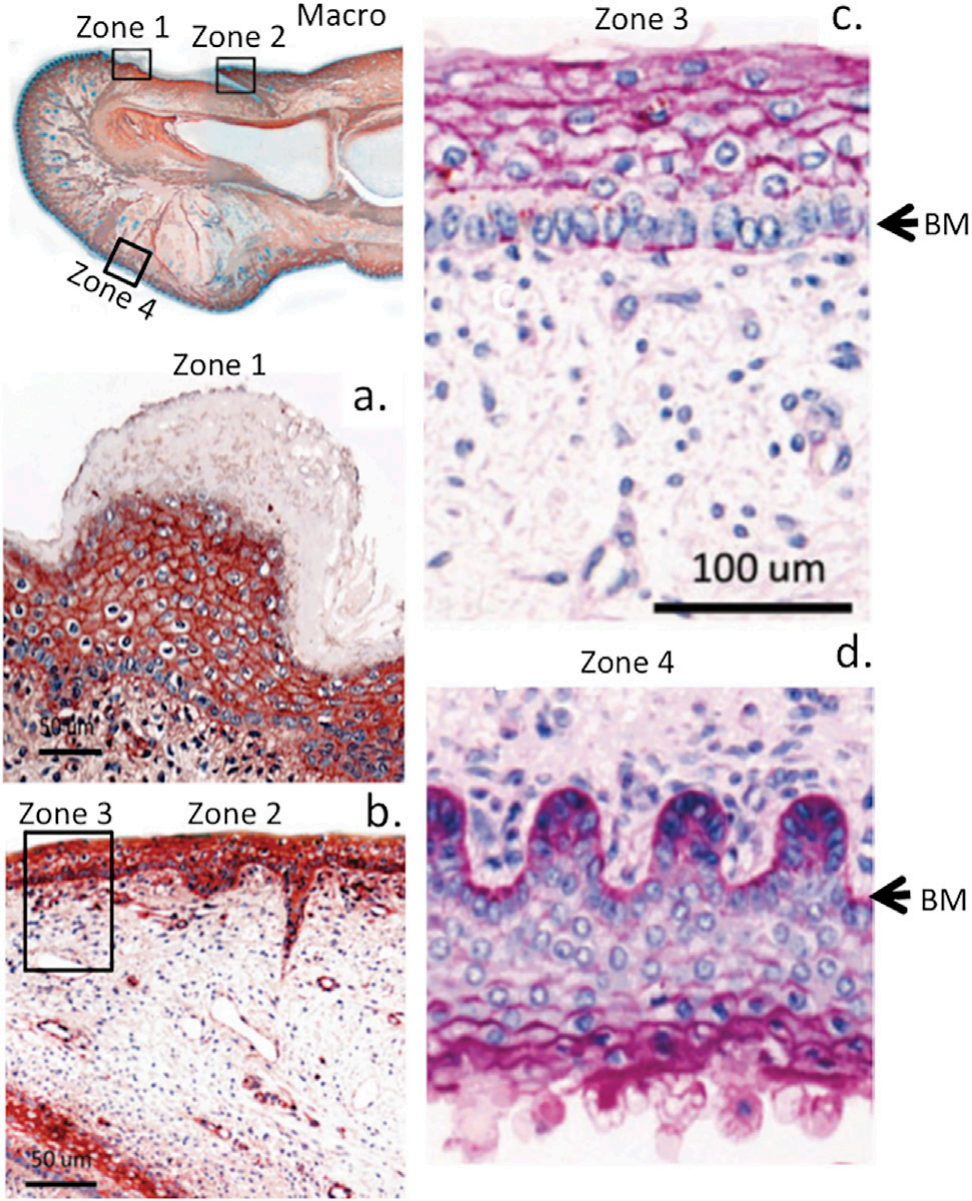
Immunolocalisation of perlecan in a 14-week gestational age human hallux (big toe). Top left panel shows a macroscopic view with boxed regions depicted at higher magnification in subjacent panels **(A,B)**. Right-hand panel shows periodic acid-schiff (PAS) staining of the foetal hallux showing selected regions of its anterior and posterior surfaces. The strong reaction (purple staining) indicates the presence of perlecan HS and other matrix polysaccharides (e.g., hyaluronic acid, HA) within the basement membrane (BM) and epidermis **(C,D)** of the digit. Images modified from (Smith and Melrose, 2015) with permission. Images © the authors 2015.

Perlecan is a large multi-domain ECM HSPG detectable in four cell blastocysts during embryogenesis, its expression changes spatiotemporally during implantation of the embryo and in the placentation stage of embryonic development (Smith et al., 1997). Perlecan is expressed by many embryonic cell types and tissues (Roediger et al., 2009) including the trophoblast and tropho-ectoderm, the basal lamina that underlies the uterine epithelia and endothelia, and developing decidua (Smith et al., 1997). Perlecan promotes many biological processes in the embryo during cell adhesion, growth factor binding, and modulation of apoptotic events (Girós et al., 2007; Soulintzi and Zagris, 2007). Perlecan expression and activity is controlled at the transcriptional level, through alternative splicing, and proteolytic processing of perlecan in the extracellular environment (Gomes et al., 2002). Full length perlecan acts as an extracellular scaffold supporting cell attachment, growth factor and morphogen sequestration that promoting cell proliferation and differentiation, matrix production and tissue expansion during development (Farach-Carson and Carson, 2007). Perlecan also stabilises the ECM through multi-component interactions mediated by its core protein and its GAG chain substitution in domain I. Perlecan is a ubiquitous modular instructive multifunctional extracellular and pericellular PG that regulates cellular migration, differentiation and proliferation (McGrath et al., 2005; Whitelock et al., 2008; Lord et al., 2014; Nakamura et al., 2015b). Perlecan in cartilaginous tissues promotes proliferation and differentiation of chondrogenic cell types, stimulates matrix synthesis and contributes to tissue expansion and skeletogenesis. It also helps stabilize the extracellular matrix (ECM) and promotes various tissue repair processes (Costell et al., 1999; French et al., 2002; Gomes et al., 2004; Sadatsuki et al., 2017; Gao et al., 2021; Garcia et al., 2021). In vascular tissues, perlecan has different effects; for example, it promotes angiogenic repair of skin wounds through FGF-2 sequestered by its domain I HS chains (Zhou et al., 2004) but inhibits smooth muscle cell (SMC) proliferation and migration (Koyama et al., 1998; Gotha et al., 2014) (Figures 3, 4).

### Perlecan’s Roles in Vascularized Tissues

In a carotid artery injury model using MΔ3/Δ3 mice lacking perlecan HS chains the medial thickness, medial area/lumen ratio, and macrophage infiltration were significantly increased (Gotha et al., 2014). Perlecan lacking HS side chains has a reduced ability to inhibit SMC proliferation *in vitro*. The HS side chains of perlecan have critical roles to play in the vessel wall, as an interleukin (IL)-2 receptor for vascular SMCs (Arumugam et al., 2019). IL-2, a 15 kDa immunoregulatory cytokine secreted by T cells, promotes peripheral immune cell growth and development initiating a defensive immune response as an initial response in the wound repair process. While SMC proliferation is inhibited by HS-PGs, digestion of HS chains from perlecan reverses this effect. SMC perlecan is a hybrid HS/CS PG while endothelial perlecan is mono-substituted with HS (Whitelock et al., 2008; Lord et al., 2014; Melrose, 2020). SMCs bind to perlecan core protein only when perlecan’s GAG side chains have been removed. This involves a novel binding site in perlecan domain III, perlecan domain V and α2β1 integrin. Endothelial cells, however, adhere to perlecan core protein containing intact GAG substitution (Whitelock et al., 1999; Lord et al., 2014).

SMC perlecan binds FGF-1 and FGF-2 via HS side chain interactions promoting FGF-2, but not FGF-1 signaling (Lord et al., 2014). Endothelial cell perlecan also binds both FGF isoforms through HS but, in this case, promotes signaling through both FGF-1 and FGF-2. Perlecan differentially regulates cellular proliferation and cell signaling promoted by growth factors to regulate tissue repair processes (Segev et al., 2004; McGrath et al., 2005; Hultgårdh-Nilsson and Durbeej, 2007). The form of perlecan present in tissues can vary with different cell populations assembling variable GAG side chain components in domain I. Perlecan is also subject to post-translational modifications including truncations, mis-sense and point mutations in its core protein and proteolytic modifications leading to the generation of perlecan fragments (Melrose, 2020). Domain IV of full-length perlecan is particularly susceptible to cleavage by matrix metalloproteinases (MMPs) leading to the generation of bioactive domain I and V matricryptic fragments.

Attachment of perlecan to the endothelial cell surface as a dynamic flow sensor at the endothelium-blood interface (Siegel et al., 2014) can also modulate charge density at the endothelial cell surface affecting membrane polarization (Siegel et al., 2014). Shear stress signaling to endothelial cells regulates vascular ECM remodeling and induction of angiogenesis (Russo et al., 2020). Membrane polarization regulates cell proliferation, cell signaling, cytoskeletal organization and gene expression (Gradilla et al., 2018). Cell polarization facilitates cell-cell signalling and is interfaced with stimulatory biophysical forces at the cell surface that regulate cell differentiation and tissue development (Saha et al., 2018). Calcium signalling initiated through transient receptor potential (TRP) endothelial cell channels (Thakore and Earley, 2019) drives vasculogenesis and controls the contractile properties of SMCs, vasodilation and blood pressure (Thompson et al., 2011). Perlecan domain II is a LDL receptor that facilitates lipid clearance from the circulation (Ebara et al., 2000). Membrane de-polymerization resulting from binding of LDL to perlecan leads to vasoconstriction, lowering of cyclic guanosine monophosphate (cGMP) SMC levels and deleteriously contributes to atherosclerosis. Lipid binding to perlecan in bone may also modulate its flow sensory properties and the regulatory properties it conveys to osteocytes (Thompson et al., 2011; Wang, 2018).

### Perlecan’s Roles in Cartilaginous Tissues

Perlecan is localized in the periphery of stem cell niches in foetal cartilage rudiments (Melrose and Melrose, 2016) (Figures 2B–D). Perlecan regulates the attainment of stem cell pluripotency and the development of migratory chondroprogenitor stem cell lineages with roles in diarthrodial joint development, expansion of the cartilage rudiments and development of primary and secondary ossification center precursors to the cartilage growth plate cartilages. These are important features of relevance in potential repair applications that might be developed using perlecan in repair biology.

While perlecan is a component of basement membranes in vascularised tissues it also has a wide distribution in tensional and weight bearing cartilages such as the meniscus, tendon, ligament and intervertebral disc (IVD). These are predominantly avascular tissues devoid of a basement membrane, however the PCM of chondrocytes has been suggested to represent an intrinsic basement membrane around each cell (Kvist et al., 2008). Atomic force microscopy (AFM) studies demonstrate that perlecan imparts compliancy to the PCM and is cytoprotective (Guilak et al., 2021). Cell-ECM interconnections in cartilages and perlecan as a biosensor, facilitates cell-matrix communication allowing cells to perceive and respond to perturbations in their biomechanical microenvironments and to orchestrate tissue homeostasis. Perlecan also monitors the flow of cannalicular fluid in the osteocyte PCM and acts as a mechanosensor that regulates bone development (Thompson et al., 2011; Wang et al., 2014; Wijeratne et al., 2016).

### Perlecan’s Roles in Neural Tissues

**FIGURE 5:**
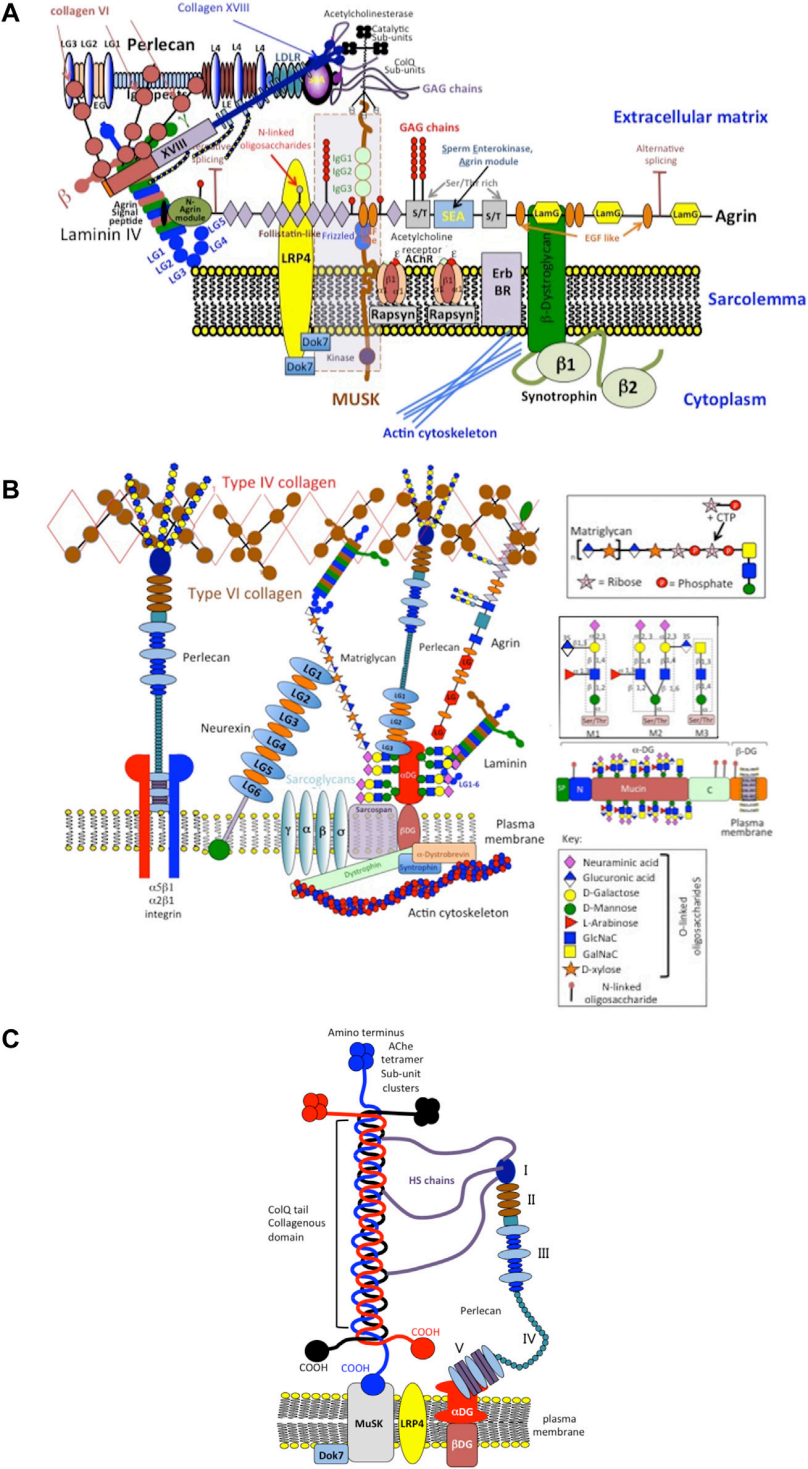
Schematic depiction of perlecan’s role as an integral structural component involved in the assembly and function of the neuromuscular junction (NMJ) showing its interactions with cell surface integrins, type IV, VI, XVIII, ColQ collagens, MuSK (Muscle-Specific Kinase) and dystroglycan (DG) and localization of catalytically active acetylcholinesterase sub-units **(A)**. Other structural components of the NMJ include extracellular components such as matriglycan, neurexin, laminin, type XVIII collagen and agrin and the cell membrane components MuSK, sarcoglycan, dystrophin and sarcospan **(B)**. This schematic is a simplified interpretation of data from the many publications that have outlined the very complex structure and function of the NMJ (Geppert et al., 1992; Crosbie et al., 1999; Arikawa-Hirasawa et al., 2002b; Steen and Froehner, 2003; Rotundo et al., 2008; Sigoillot et al., 2010; Knight et al., 2011; Singhal and Martin, 2011; Ohno et al., 2013; Arredondo et al., 2014; Yoshida-Moriguchi and Campbell, 2015; Banerjee et al., 2017; Cescon et al., 2018; Belhasan and Akaaboune, 2020; Legay and Dobbertin, 2020) and illustrates how mutations in perlecan evident in SJS that result in severely diminished tissue levels of perlecan compromise the functional properties of the NMJ manifesting in the neurological and muscular deficits evident in SJS. A better understanding of the functional basis of the NMJ is also of relevance to synaptic functions in musculoskeletal disorders in general. Perlecan has key roles to play in the assembly, function and regulation of the NMJ (Aldunate et al., 2004; Cartaud et al., 2004; Kimbell et al., 2004; Rotundo et al., 2005; Rudenko, 2017; Südhof, 2018; Noborn and Sterky, 2021). Perlecan has central roles in the clustering of acetylcholinesterase (Ache) at the synaptic basal membrane through formation of a ternary complex with MuSK, DG and ColQ **(C)**. The collagen-tailed form of AChE is localized at the NMJ through interaction with the transmembrane DG complex by binding to perlecan (Kimbell et al., 2004). HS binding domains in ColQ anchor it to the synaptic basal lamina. ColQ-AChE/perlecan complex co-localizes in the NMJ with dystroglycan, rapsyn, laminin and MuSK (Rotundo et al., 2005). MuSK is a receptor tyrosine kinase with important roles to play in the clustering of active AChE sub units at the NMJ in a ternary complex with ColQ and perlecan (Cartaud et al., 2004) of functional importance (Aldunate et al., 2004). Neurexin HS chains also recruit HS-binding proteins required for synaptic assembly and the maintenance of synaptic plasticity (Südhof, 2018; Noborn and Sterky, 2021). A large collection of synaptic adhesion/organizing molecules (SAMs) exist in the mammalian brain with roles in synapse development and maintenance. SAMs, include neurexins, neuroligins, cadherins, and contactins implicated in neuropsychiatric and neurodevelopmental diseases, including autism, schizophrenia, and bipolar disorder (Rudenko, 2017). A greater understanding of the process of synaptic assembly, function and regulation at the molecular level may further the development of novel synaptic therapeutics. A recent publication proposes that HS-PGs are key players in Alzheimer’s disease (AD). In a unifying hypothesis HS-PGs are considered central to all aspects of AD neuropathology, i.e., plaque/tangle development amyloid deposition, neuroinflammation and apolipeprotein E (ApoE) accumulation (Snow et al., 2021).

**FIGURE 6:**
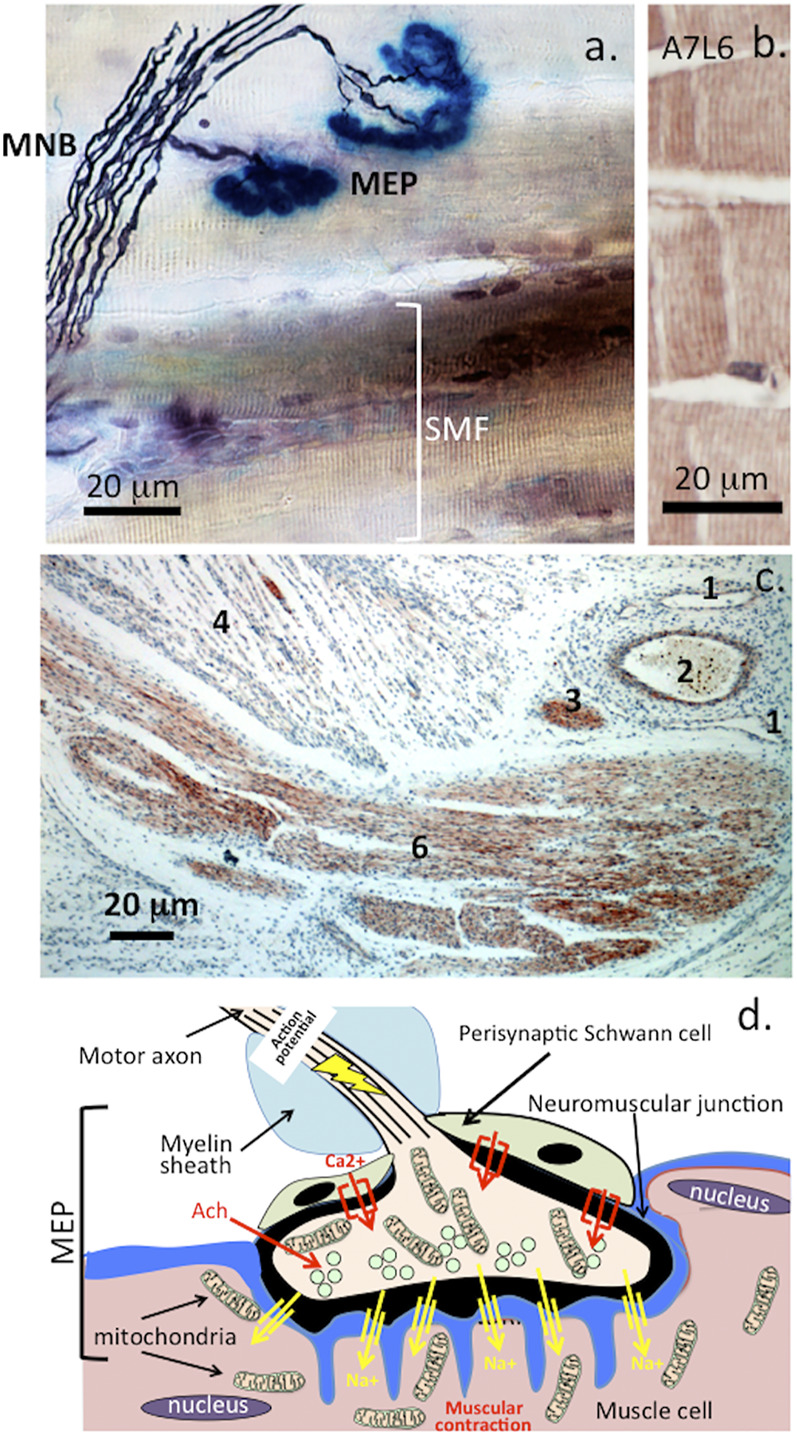
Histochemical localization of a motor neuron attaching to muscle fibre showing the myelinated nerve bundle (MNB), motor endplates (MEP) and striated muscle fibres (SMF) **(A)**. and perlecan-positive striated muscle fibres stained with MAb A7L6 to perlecan domain IV in murine extensor digitorum longus muscle **(B)**. A lower power image depicting perlecan-positive features in a foetal human elbow joint **(C)**. 1) flattened venule, 2) capillary with entrapped red blood cells, 3) small nerve -bundle in cross-section, 4) muscle fibres, 5) longitudinal nerve fibre bundles in ulnar nerve of the elbow. Schematic of the major features of the motor end plate **(C)**. Figure segment a, stock image 2AD3P00 from Alamy Science Photolibrary reproduced under license. **(B,C)** modified from (Shu et al., 2019) Open Access under CC BY NC-ND License to Publish. **(D)**. A wave of membrane depolarization emanating from the nerve soma produces an action potential that travels down the nerve axon resulting in activation of voltage gated Ca2+ channels in the nerve synapse and a resultant influx of Ca2+. This results in mobilization of synaptic vesicles in the nerve to the post synaptic membrane. These merge with the post-synaptic membrane releasing their neurotransmitter contents which include acetylcholine into the synaptic gap. Acetylcholine is captured by acetylcholine receptors on the muscle synaptic membrane which results in an influx of Na+ ions through Na+ channels into the muscle causing muscular contraction. Acetylcholinesterase released by the nerve mops up any excess of acetylcholine. Acetylcholinesterase is one of nature’s most efficient enzymes and hydrolyses acetylcholine regulating muscular relaxation (Vigny et al., 1978). The myotonia found in SJS is due to a breakdown in this mechanism due to a deficiency of perlecan at the NMJ and a deficiency of AChE that is normally clustered in synapses by perlecan (Silman and Sussman, 2008).

Perlecan has critical roles in basement membrane in the blood brain barrier and important roles in NMJ assembly and function (Figures 5, 6). Perlecan-FGF-2 interactions in the neural stem cell niche (fractone) regulate the survival, proliferation and differentiation of neuroprogenitor stem cell populations in the sub-ventricular and dentate gyrus of the hippocampus (Kerever et al., 2007; Douet et al., 2013; Kerever et al., 2014; Mercier, 2016; Kerever and Arikawa-Hirasawa, 2021; Kerever et al., 2021; Kim et al., 2021).

## Perlecan Mutations and Animal Models: Functional Clues They Provide on the Biological Roles of Perlecan

### Schwartz-Jampel Syndrome (Chondrodystrophic Myotonia) and Dyssegmental Dysplasia Silverman-Handmaker Type

The importance of perlecan to the functional weight bearing and tensile properties of cartilaginous tissues is well illustrated in SJS (Schwartz-Jampel syndrome; chondrodystrophic myotonia) (Arikawa-Hirasawa et al., 2002a) and Dyssegmental dysplasia Silverman-Handmaker type (DDSH) (Arikawa-Hirasawa et al., 2001a; Arikawa-Hirasawa et al., 2001b). The former is a relatively mild skeletal condition characterized by reduced perlecan levels in tissues; however, DDSH is a very severe condition where perlecan levels in tissues are severely depleted or totally absent. SJS is an autosomal recessive disease caused by mutation in the *HSPG2* gene resulting in skeletal dysplasia and neuromuscular hyperactivity (Nicole et al., 2000; Bauché et al., 2013). In this condition mutant fibroblasts secrete reduced levels of perlecan and display impaired migratory properties but normal proliferative rates (Arikawa-Hirasawa et al., 2002a). DDSH (MIM 224410) in contrast is a very severe but extremely rare condition caused by functional null mutations in the perlecan HSPG2 gene (Arikawa-Hirasawa et al., 2001a). Less than forty DDSH cases have been reported in the literature, and only four of these were detected prenatally.

### Perlecan Knockout Model

Perlecan knockout is a lethal condition. Conventional perlecan knockout (i.e., Hspg2^−/−^, KO) mice die just after birth mainly due to respiratory failure (Arikawa-Hirasawa et al., 1999; Costell et al., 1999). The few mice that survive to birth display macroscopic abnormalities in cephalic development and have short squat frames with severely distorted axial and appendicular skeletal development. Furthermore, internal examination shows major abnormalities in the development of the major outflow tracts from the heart in these mice. Perlecan-null mice, form normal basement membranes but these soon deteriorate at areas of increased mechanical stress e.g., areas of myocardial contraction and brain vesicle expansion. Perlecan-null mice die around E10–12, due to heart, lung, and brain defects. Weakened embryonic heart basements membranes and “leaky” cardiomyocyte-endothelial cell interfaces result in cardiac arrest due to blood leakage into the pericardial space. Major defects in lung development also contribute to the lethality of perlecan deficiency. Abnormalities in cephalic development, distortion in normal brain laminar architecture and development of holes in the fore- and midbrain also occur. Perlecan-null mice experience severe bleeding in the lung, skin, and brain, due to weakened blood vessels. Perlecan null mice also display distorted growth plate architecture and a disturbed chondrocyte organization with a loss of normal columnar chondrocyte spatial organization and expansion and distortion of the resting, proliferative and hypertrophic zones consistent with the massive disruptions seen macroscopically in skeletal development in this mouse model. Perlecan knock-out mice are not suitable for the examination of perlecan’s roles in postnatal tissue development, but starkly demonstrate the importance of perlecan in the development of pre-natal vascular and non-vascular tissues.

### Perlecan Conditional Transgenic Model

In order to avoid the lethality of perlecan KO mice, conditional perlecan-deficient (Hspg2^−/−^, TG) mice were developed that express the perlecan transgene only in cartilage using the Col2a1promoter and enhancer (Xu et al., 2010; Xu et al., 2016). These perlecan TG mice develop a phenotype similar to DDSH but also share features of SJS (Arikawa-Hirasawa et al., 2001a; Arikawa-Hirasawa et al., 2002a). Perlecan knockdown impacts on cartilage development and skeletogenesis and impairs the normal weight bearing properties of cartilaginous tissues and functional properties of the pericellular matrix of chondrocytes (Xu et al., 2016; Ocken et al., 2020).

### Perlecan Exon 3 Null (HS Deletion) Model

In Hspg2^−/−^-Tg (Hspg2^−/−^; Col2a1-Hspg2^Tg/−^) mice, perlecan is only expressed in cartilage but not the synovium. This results in less development of osteophytic spurs in the tibial and femoral joint margins (Arikawa-Hirasawa et al., 1999; Xu et al., 2010; Ishijima et al., 2012; Kaneko et al., 2013). A perlecan exon 3 null mouse model has also been developed. Perlecan domain-I encoded by exon 3 contains its GAG attachment points thus perlecan exon 3 null mice produce perlecan deficient in HS. A 20 kDa drop in the size of the perlecan core protein is also evident. This model has yielded important information on the role of perlecan HS in the regulation of chondrocyte behavior and in tissue homeostasis and perlecan HS mediated interactions with ECM components (Whitelock et al., 2008; Smith et al., 2010; Hayes et al., 2011a; Hayes et al., 2011b; Hayes et al., 2013; Shu et al., 2013; Hayes et al., 2014; Shu et al., 2016a; Hayes et al., 2016b; Shu et al., 2018; Shu et al., 2019; Smith and Melrose, 2019). This exon 3 null GAG free form of perlecan does not participate in growth factor and morphogen sequestration and cell signaling like the full length GAG substituted form of perlecan resulting in a loss in its ability to act as a co-receptor for the presentation of these growth factors to their cognate receptors (Zhou et al., 2004; Whitelock et al., 2008). However, there are GAG-free regions of the perlecan core protein that can also bind certain growth factors. For example, perlecan domain III can bind FGF-7 and FGF-18, however it is not known to what extent this interaction can provide the same cell proliferative and differentiative properties provided by growth factors that bind to the GAG chains of perlecan domain I. Perlecan exon 3 null HS-deficient mice do not store TGF-β1 in skin tissues like the full-length perlecan does (Shu et al., 2016a). Participation in the wound healing response, previously provided by FGF-2 and VEGF perlecan interactions and the angiogenic responses they provide, is also lost in perlecan exon 3 null mice (Zhou et al., 2004). As previously discussed, full length HS substituted perlecan has major roles in tissue and organ development and wound healing orchestrated by the binding and signaling of mitogens and morphogens with cells in a temporal and dynamic fashion. FGF-7, −18 and PDGF can also bind to perlecan domains III, IV and V, such interactions may also mediate wound healing and cell signaling responses. Binding of PDGF-BB has been mapped to domain III-2 (Kd = 8 nM), lower binding affinities are evident for domains I, IV-1 and V (Kd = 34–64 nM). PDGF-AA binds to domain III-2 (Göhring et al., 1998). Perlecan HS deficiency impairs pulmonary vascular development (Chang et al., 2015). Perlecan HS chains also recruit pericytes to pulmonary vessels. HS deficiency in perlecan attenuates hypoxia-induced pulmonary hypertension involving impaired FGF-2/FGFR1 interactions (Chang et al., 2015). Perlecan exon 3 null mice display reduced healing responses due to impaired FGF-2 and VEGF signaling (Zhou et al., 2004). The chondroprotection evident in perlecan exon 3 null mice in a post traumatic OA model may be attributable to the preservation of FGFR-3-FGF-18 signaling and perlecan domain III-mediated interactions (Shu et al., 2016b), contributing to significantly reduced joint margin osteophytosis, synovial perlecan is required for osteophyte formation in knee OA (Kaneko et al., 2013). *Hspg2* exon 3 null murine chondrocytes display increased hypertrophic maturational changes and chondrocyte proliferative rates *in vitro* and *in vivo*, accelerated growth plate maturation, elevated GAG deposition, and exostosis formation in the IVD (Shu et al., 2019). Perlecan HS may thus exert repressive control over chondrocytes in mature cartilage explaining why cartilage has such a poor healing response (Garcia et al., 2021).

### Schwartz-Jampel Syndrome (Chondrodystrophic Myotonia) Model

A model of SJS in which a 4595G to A point mutation occurs in the perlecan gene displays reduced perlecan secretion and incorporation into the PCM similar to the clinical features of human SJS (Rodgers et al., 2007; Stum et al., 2008). Mice homozygous for Hspg2C1532Y-Neo (Neo/Neo) have short stature, impaired mineralization, misshapen bones, OA-like joint dysplasias and myotonia (Rodgers et al., 2007; Stum et al., 2008).

A model of SJS has also been developed that displays congenital peripheral nerve hyper-excitability, neuromyotonia, demyelination and peripheral neuropathies (Bangratz et al., 2012). This model revealed roles for perlecan in the regulation of longitudinal elongation of myelin by Schwann cells (Echaniz-Laguna et al., 2009; Bangratz et al., 2012). Perlecan-deficient mice displaying shorter internodes, had increased levels of impaired functional voltage-gated K (+) channels (Echaniz-Laguna et al., 2009). Electrophysiological studies have demonstrated muscle fiber hyper-excitability arising from such alterations in peripheral nerve organization, muscle hypertrophy and compositional changes (Xu et al., 2010).

### Perlecan Cerebral Artery Occlusion Stroke Model

Cerebral artery occlusion stroke models in Yucatan miniature pigs, dogs and mice (Platt et al., 2014; Vasquez et al., 2019; Llovera et al., 2021) have facilitated examination of perlecan’s roles in the repair of the blood brain barrier following stroke. Perlecan domain V has neurogenic and neuroprotective properties and promotes angiogenic repair of the blood brain barrier (Lee et al., 2011; Bix, 2013; Marcelo and Bix, 2014). Perlecan transgenic mice have demonstrated important roles for perlecan domain V in pericyte recruitment in the promotion of BBB repair processes (Nakamura et al., 2019). IL-1 is also neuroprotective and has neuron restoring properties in experimental ischemic stroke studies (Salmeron et al., 2019). Recombinant perlecan domain V is now available for blood brain barrier repair strategies (Rnjak-Kovacina et al., 2017). Recombinant perlecan domain V decorated with HS and CS chains is a vascular PG in its own right and supports endothelial cell interactions as well as full-length perlecan (Rnjak-Kovacina et al., 2017). Repair of the blood brain barrier involves pericyte recruitment, triggered by an up-regulation in PDGFRβ (Arimura et al., 2012; Makihara et al., 2015; Shibahara et al., 2020), this drives pericyte migration required for pericyte endothelial tube repair interactions in the neurovascular unit (Hellstrom et al., 1999). Perlecan binds PDGF and promotes pericyte migration and integrin α5β1 and α2β1 mediated interactions in endothelial tube formation (Hellstrom et al., 1999; Arimura et al., 2012; Shen et al., 2012; Makihara et al., 2015; Nakamura et al., 2019).

## Application of Perlecan in Repair Biology

### Cell-Mediated Effects of Perlecan: Modulation of Cell Proliferation

While perlecan promotes the proliferation and differentiation of endothelial cells, as well as many other cell types, it inhibits SMC proliferation through the tumor repressor PTEN (tumor suppressor phosphatase and tensin homolog), including upregulation of FRNK (focal adhesion kinase–related non-kinase) and down regulation of FAK signalling (Walker et al., 2003; Garl et al., 2004).

### Perlecan’s HS Mediated Interactions and Their Relevance to Tissue Repair

HS’s interactions with FGF-2, PDGF, VEGF, HGF, BMP2, GM-CSF, angiopoietin-3, and activin A illustrate the potential of perlecan domain I as a co-receptor for growth factor delivery and receptor activation. The ITIM megakaryocyte-platelet receptor (G6b-B-R) is an additional ligand for the HS chains of perlecan domain I (Vögtle et al., 2019). G6b-B-R is highly expressed in mature megakaryocytes (MKs) that regulates platelet activation (Lord et al., 2018; Vögtle et al., 2019). Binding of G6b-B-R to perlecan HS chains mediates functional responses in MKs and platelets, negatively regulating platelet adhesion to fibrinogen and collagen. It also modulates platelet adhesion to vascular graft materials and explains the varied roles of perlecan in fibrosis (Lord et al., 2009; Lord et al., 2018).

### Understanding Perlecan’s Cell Regulatory Roles in Blood Vessels

Perlecan attached to endothelial cells in the lumen of blood vessels acts as a shear flow sensor that interacts with Ca2+ or Na+ regulating charge density at the endothelial cell surface. This also regulates membrane polarization in endothelial cells and SMCs, ion transport regulates vasoconstriction and relaxation in blood vessels (Siegel et al., 2014) and is crucial for cell-cell signalling coupled with stimulatory biophysical forces that promote cell differentiation and tissue development (Saha et al., 2018). Calcium signalling through endothelial cell TRP channels (Thakore and Earley, 2019) drives vasculogenesis (Moccia et al., 2019) and SMC contractility which in turn regulates vasodilation and blood pressure (Ishai-Michaeli et al., 1990).

### Heparanase has Roles in Wound Repair and Tissue Remodelling

While the HS chains of perlecan mediate growth factor interactions in skeletogenesis, degradation of HS *in situ* has been shown to improve wound healing through the re-mobilization of sequestered growth factors locally at sites of tissue repair (Ishai-Michaeli et al., 1990; Zcharia et al., 2005; Nasser, 2008). Heparanase expression in osteoblastic cells also promotes bone formation and tissue remodeling at the osteochondral interface during endochondral ossification (Kram et al., 2006).

### Application of Perlecan in Vascular Tissue Repair

Given the crucial role that perlecan plays in multiple biological processes, it is unsurprising that perlecan, or its components, have been utilized in multiple therapeutic applications. Perlecan’s known ability to bind, sequester, and deliver a myriad of growth factors and other bioactive molecules is a key feature that has been harnessed to translate this molecule into potential therapeutics for multiple applications, from angiogenesis to the regeneration of cartilage (Gao et al., 2021; Garcia et al., 2021). Perlecan’s ability to modulate processes in cardiovascular applications has been explored through an immune-purified form of perlecan from human coronary arterial endothelial cells. This immunopurified perlecan was used to coat expanded polytetrafluoroethylene (ePTFE) vascular grafts. Implantation of the vascular grafts into the carotid arteries of an ovine model demonstrated that the perlecan-coated grafts reduced platelet adhesion and enhanced endothelial cell growth along the implanted graft when compared with the uncoated vascular graft (Lord et al., 2009). While this study demonstrated the ability for perlecan to improve vascular graft patency, isolation of perlecan from tissues or purified from conditioned medium, can be cost-prohibitive due to the small amounts available and the quantities required.

An alternative option that has been explored is through the use of recombinant fragments of perlecan. The protein core of perlecan contains five domains, many with bioactive properties, though use of recombinant perlecan fragments has focused on the use of domain-I and -V due to these domains containing GAG attachment sites. The protein component of perlecan domain I contains three GAG attachment sites. The GAGs that decorate the perlecan domain I core protein are predominantly HS, though it may also be decorated with CS. Recombinant perlecan domain I decorated with HS has been incorporated into 3D structures or scaffolds for multiple therapeutic applications. The incorporation of perlecan domain-I into 3D structures has resulted in increased retention of FGF-2 (Yang et al., 2005), as well as BMP2 (Jha et al., 2009; Srinivasan et al., 2012; Chiu et al., 2016) for cartilage repair and regeneration. More recently, advances in fabrication and microfluidics has enabled the ability to produce growth factor gradients (Hubka et al., 2019), an approach that has been utilized to generate chemotactic gradients with FGF-2R The ability to create gradients using perlecan, perlecan fragments and other bioactive components of the ECM, has significant potential in tissue repair and regeneration, including the development of smart biomaterials and constructs. It will also greatly improve the understanding of many developmental and disease processes, e.g., in cancer biology.

As mentioned above, perlecan domain V, like domain I, contains a GAG attachment site. Recombinant perlecan has been explored in several applications due to its growth factor interactions. Recombinant perlecan domain V is substituted with HS and CS chains and promotes angiogenesis by enhancing growth factor signaling (Lin et al., 2020). When perlecan or perlecan DNA is immobilized on silk or chitosan scaffolds (Lord et al., 2017) increased vascular ingrowth and integration *in vivo* underlies the importance of perlecan in angiogenesis/vasculogenesis.

The complexity and nuances of perlecan’s ability to modulate biological processes has been explored by immobilization technique, and the presence of GAG chains plays a key role in the orientation of this PG (Rnjak-Kovacina et al., 2016). Immobilization of perlecan domain V by physisorption or covalently, in addition with immobilization of the protein core of perlecan domain V only, or protein core decorated with GAG chains, modulated the interaction and attachment of both endothelial cells and platelets. The ability to control the interaction of different cell types holds tremendous importance to tissue repair, for example in cardiac and vascular repair procedures where tissue grafts and implants, require cues to facilitate migration and re-endothelization of the repair tissue, whist minimizing platelet adhesion. The potential of perlecan domain V to modulate platelet adhesion has been explored by incorporating this domain onto different polymeric surfaces including poly (vinyl chloride) (Chandrasekar et al., 2021), silk (Lau et al., 2021) and chitosan (Lord et al., 2017). Incorporation of perlecan domain I plasmid DNA in conjunction with VEGF189 in a rodent wound model demonstrated increased re-epithelialization of the wound, formation of sub-endothelial tissue and neo-angiogenesis within the wound bed (Lord et al., 2017).

### Application of Stimulatory Peptides Derived From ECM Components in Repair Biology*: In Vitro* Engineering of Salivary Glands Using Perlecan Domain IV, Laminin-111 and Fibronectin Peptide Scaffolds

Peptides derived from perlecan domain IV (TWSKV), laminin-111 (YIGSR, IKVAV), and fibronectin (RGDSP) have been incorporated into HA scaffolds with RGDSP and TWSKV peptide HA scaffolds significantly accelerating cell proliferation (Fowler et al., 2021). Perlecan peptide TWSKV triggers differentiation of salivary gland cells into self-assembling acini-like structures expressing salivary gland biomarkers that secrete α-amylase (Pradhan et al., 2009; Pradhan et al., 2010). Purified ECM-derived peptides have been suggested as stimulatory molecules that direct the proliferation and differentiation of progenitor cell populations. These are of potential application in tissue repair processes using human embryonic stem cells (hESCs) and induced pluripotent stem cells (iPSCs) (Rowland et al., 2013). Laminin-111 peptide fibrin hydrogels restore salivary gland function (Nam et al., 2017). An extensive range of laminin-111-derived peptides conjugated to chitosan scaffolds also show promise in tissue engineering applications designed to promote tissue regeneration (Hozumi et al., 2012). Laminin-111 peptide-HA hydrogels have also been shown to act as a synthetic basement membrane (Yamada et al., 2013). Conjugation of RGDSP peptide to HA gels improves cell viability, accelerates formation of epithelial spheroids, and promotes the expansion of 3D progenitor cell populations, representing the first step toward the development of an engineered salivary gland (Fowler et al., 2021). Primary salivary human progenitor stem cells undergo acinar-like differentiation in HA hydrogel cultures and incorporation of basement membrane peptides derived from perlecan and laminin-111 further directs the development of 3D salivary gland-like spheroids (Srinivasan et al., 2017). Full-length perlecan also has directive roles over the 3D development of submandibular salivary glands. Heparanase colocalizes with perlecan in submandibular gland basement membrane. Cleavage of perlecan HS side chains by heparanase regulates salivary gland branching morphogenesis by modulating FGF-10 mediated cell signaling. Heparanase releases FGF-10 from perlecan HS in the basement membrane. This increases MAPK signaling, epithelial clefting, and lateral branching thus increasing submandibular gland branching morphogenesis (Patel et al., 2007). The size and sulfation patterns of the perlecan HS side chains regulate FGF-10-mediated interactions during proliferation, salivary gland duct elongation, bud expansion, and differentiation. The spatio-temporal localization of specific HS structures in salivary tissues provides a mechanistic insight as to how salivary gland developmental processes mediated by FGF10 occur *in vivo* (Patel et al., 2008). FGF-10 is a multi-functional paracrine growth factor that mediates mesenchymal-epithelial signaling during tissue development, growth and disease and has significant relevance to regenerative medicine (Itoh and Ohta, 2014; Itoh, 2016).

### Potential Roles for Perlecan in Cartilage Repair

Once damaged, articular cartilage has a notoriously poor ability to repair itself (Armiento et al., 2019). Perlecan’s important well-established roles in chondrogenesis and cartilage development (Smith et al., 2010; Melrose et al., 2012) imply potential roles in cartilage repair by recapitulating developmental roles in damaged/diseased tissues (Gao et al., 2021; Garcia et al., 2021). Perlecan promotes chondroprogenitor stem cell maturation and development of pluripotent migratory stem cell lineages with roles in diarthrodial joint formation and early cartilage development (Hayes et al., 2016a; Melrose and Melrose, 2016). Perlecan’s cartilage stabilizing properties through interactions with ECM components also establish its potential in cartilage repair (SundarRaj et al., 1995; Gomes et al., 2002; Melrose et al., 2006; Melrose et al., 2008). Perlecan domain I hydrogels have been used to establish heparin binding growth factor gradients that promote cell migration of potential use to promote cartilage repair (Hubka et al., 2019). Perlecan domain I BMP-2 hydrogels have also been shown to promote chondrogenesis and cartilage repair in a murine early OA model (Yang et al., 2006; Jha et al., 2009; Srinivasan et al., 2012). BMP-2 and BMP-9 can also promote chondrogenic differentiation of human multipotential mesenchymal stem cells (Majumdar et al., 2001; Shestovskaya et al., 2021). Perlecan domain I collagen I fibril scaffolds have been used as an FGF-2 delivery system that could also be useful in cartilage repair strategies (Yang et al., 2005). FGF-2 directs non-committed mesenchymal stem cells to a chondrogenic phenotype (Shu et al., 2016c).

### Roles for Perlecan at the Neuromuscular Junction

The duration of synaptic transmission of impulses at the NMJ is chiefly controlled by acetylcholinesterase (AChE), an enzyme which hydrolyzes acetylcholine (ACh) to choline and acetate (Katz and Miledi, 1973; Rosenberry, 1979). Perlecan, nidogens, glycoproteins and collagen fill the synaptic cleft and are relevant for signal transduction as well as the maintenance of mechanical strength in the NMJ. At the NMJ, the most abundant form of AChE is the AChE_T_ which is anchored to the synaptic basal lamina by non-fibrillar collagen Q (ColQ) (Krejci et al., 1991; Krejci et al., 1997). ColQ, a triple helical structure, binds to perlecan domain 1 via two heparan sulfate PG binding motifs and to muscle specific kinase (MuSK) respectively (Rotundo et al., 1997; Casanueva et al., 1998; Deprez et al., 2003; Cartaud et al., 2004; Nakata et al., 2013).

Experimental reports demonstrate the functional relationship between perlecan and AChE (Peng et al., 1999; Arikawa-Hirasawa et al., 2002b; Massoulié and Millard, 2009; Maselli et al., 2012) and the cellular co-localization of perlecan and AChE at the synaptic basal lamina *in vitro* and in pre-clinical models of the NMJ (Peng et al., 1999; Arikawa-Hirasawa et al., 2002b; Massoulié and Millard, 2009; Maselli et al., 2012). Perlecan null mice lack AChE clusters at the NMJ (Peng et al., 1999; Martinez-Pena y Valenzuela et al., 2007).

### Perlecan and Stroke

Ischemic stroke is a leading cause of permanent disability and mortality and there is an urgent need for adjunctive therapies for concurrent administration with existing clot-busting (tissue plasminogen activator, tPA) and endovascular clot removal (thrombectomy) treatments. Whilst these primary treatments remove the underlying cause of the stroke, they do little to reverse or prevent subsequent brain injury. Perlecan domain V is potentially one such therapy whose efficacy has been experimentally demonstrated in both young and aged rodent models of ischemic stroke, where it has been reported to have neuroprotective, neuroreparative and functionally restorative effects (Lee et al., 2011; Bix et al., 2013; Trout et al., 2021). These benefits of perlecan domain V have been linked to its maintenance of the BBB through α5β1 integrin modulation and increased VEGF secretion (Clarke et al., 2012; Nakamura et al., 2019). Bix and colleagues showed that brain expression of perlecan is substantially and chronically increased following experimental and clinical ischemic stroke (Lee et al., 2011; Bix et al., 2013; Trout et al., 2021). This observation has led to the hypothesis that perlecan plays a significant role in brain repair following ischemic insult. Accordingly, larger infarct volumes and severe motor-sensory deficits in transgenic mice with only 10% of total perlecan bioavailability (perlecan hypomorphs), when compared to age-matched wild type controls, have been independently reported and corroborated (Lee et al., 2011; Nakamura et al., 2019). This suggests that physiologic levels of perlecan are important for the brain to repair itself after stroke, and that diminished perlecan levels are detrimental for stroke outcomes. It follows that exogenously administered perlecan domain V would enhance the brain’s ability in self-repair by increasing the availability of perlecan for BBB maintenance, thereby improving brain injury and functional deficits following ischemic stroke.

Perlecan promotes blood brain barrier repair following acute brain injury by modulating the survival and migration of PDGFRβ-positive pericytes to the ischemic brain region (Arimura et al., 2012; Shen et al., 2012; Nakamura et al., 2016; Hoffmann et al., 2018; Nakamura et al., 2019). Perlecan domain V binds to PDGFRβ and α5β1 in order to maintain blood brain integrity in the injured brain area (Nakamura et al., 2019) Also, following ischemic brain injury, α5β1 integrin expression increases and the exogenously administered perlecan domain V binds to this integrin on endothelial cells to bring about the increased secretion of VEGF (Clarke et al., 2012). The elevated levels of VEGF enables angiogenesis *via* the downstream activation of PI3K-Akt, MEK-ERK and HIF-1α (Milner et al., 2008; Clarke et al., 2012). Surprisingly, the increased VEGF does not worsen the BBB disruption following perlecan domain V administration after ischemic stroke (Lee et al., 2011). The intraperitoneal administration of perlecan domain V decreases infarct volume as well as immuno-expression of markers of apoptosis (TUNEL and Caspase 3 cleavage) (Lee et al., 2011). More so, perlecan domain V modulates the activation and proliferation of astrocytes and ameliorates astrogliosis *in vitro* and *in vivo*. This indicates that perlecan domain V rescues neuronal death and may actively dampen scar formation after ischemic stroke suggesting it has instructive regulatory properties in tissue fibrosis (Lord et al., 2018). Matricryptin peptides derived from ECM components also have roles in tissue remodeling (Amruta et al., 2020; de Castro Brás and Frangogiannis, 2020). The *in vivo* benefits of perlecan domain V have been evaluated in rodents using various models of ischemic using middle cerebral artery occlusion which includes tandem ipsilateral common carotid and middle cerebral arteries occlusion (CCA/MCAO) model that induces mild cortical lesion (Trout et al., 2021), endothelin (ET)-1 injection (mild to moderate brain lesion) (Lee et al., 2011), photothrombotic (Bix et al., 2013) and intraluminal filament MCAO model -severe brain injury.

The therapeutic potential of exogenously administered perlecan domain V in a severe pre-clinical ischemic stroke model (photothrombotic and intraluminal MCAO) is essentially dependent on how quickly the first dose of perlecan domain V is administered after ischemic stroke induction. A recent report by Nakamura et al. (Nakamura et al., 2019) found mild effects of exogenously administered recombinant perlecan domain V (5 mg/kg) on infarct volume after intraluminal MCAO induction. Perlecan domain V was administered 24 h after reperfusion and daily for three to four consecutive days. However, Bix and colleagues had previously reported that the timely administration of perlecan domain V (2 mg/kg) after severe ischemic stroke (photothrombotic model) is vital for neuroprotective benefits (Clarke et al., 2012). Indeed, the specific report indicates that when perlecan domain V is administered 3 hours after stroke induction, infarct volume is smaller when compared to perlecan domain V administration when commenced six, twelve and 24 hours post-stroke induction. Importantly, infarct volume increases as the start of perlecan domain V treatment after stroke induction period increases. Additionally, recent findings show a significant decrease in infarct volume when perlecan domain V was co-administered during reperfusion in a mouse model of intraluminal MCAO (Biose et al., 2021).

Early reports have shown that human recombinant LG3, the C-terminal-most portion of perlecan domain V, may confer the neuroprotective efficacy of full length perlecan domain V (Saini and Bix, 2012; Bix, 2013). Like domain V, LG3 is increased in the compromised brain hemisphere after 24 hours of transient MCAO and it persistently remains elevated for 3 days after stroke in rats.^3^ Similarly, 2 hours of oxygen-glucose deprivation (OGD) in astrocytes and neuron cultures substantially increases LG3 levels. LG3 is anti-apoptotic as it decreases Caspase 3 after OGD-reperfusion (Lee et al., 2011; Saini and Bix, 2012). Also, LG3 preserved the integrity of fetal cortical neurons by reducing Caspase 3 immunohistochemistry after *in vitro* OGD when compared to control dishes (Saini and Bix, 2012). Investigations are ongoing to determine the effect of LG3 on infarct volume and motor-sensory functions in various preclinical models of ischemic stroke (Biose et al., 2021).

In summary, perlecan is actively processed for brain repair after ischemic stroke and is neuroprotective, improves angiogenesis and motor-sensory functions and modulates astrocyte activity when administered in a timely manner following stroke. Furthermore, LG3 which confers the biological activity of perlecan domain V in infarct volume reduction may have potential adjunctive stroke therapeutic value in preventing mortality among clinical stroke patients (Biose et al., 2021).

## Conclusion and Future Research

Perlecan is a ubiquitous, pleomorphic, and morphologically important PG with roles in many physiological processes in tissues of varied form and function. A greater understanding of perlecan’s tissue specific roles during growth, development and disease will be insightful as to how this multifunctional PG might be utilized to maximal effect in repair biology. The current biological understanding of perlecan demonstrates its enormous capacity as a “fix-it” molecule with many beneficial attributes (e.g., matrix stabilization, cell guidance and general tissue development) that could be potentially harnessed in repair biology. Emerging improvements in implant design, therapeutic blood brain barrier repair strategies following stroke, modeling of artificial neural stem cell niches and neuromuscular synaptic interface tissues may find application in the treatment of neurodegenerative disorders (Wang et al., 2013; Gill et al., 2015; Buzanska et al., 2018). Acetylcholine is a cholinergic agonist and chief neurotransmitter of the parasympathetic autonomic nervous system that contracts smooth muscle. Efficient regulation of acetylcholine is essential and achieved by acetyl cholinesterase located in the NMJ allowing muscle to undergo relaxation following contraction. Dysregulation of this process can have far-reaching and potentially fatal consequences with a cessation of activity of muscle groups that control breathing. As shown in this review perlecan has key roles in the assembly of the NMJ through interactions with type VI collagen (Cescon et al., 2018), Coll-Q, MuSK and dystroglycan (Peng et al., 1999) responsible for localization of acetylchinesterase in the NMJ (Rotundo et al., 2005; Rotundo et al., 2008; Rudenko, 2017). Acetylcholinesterase is one of the most rapidly acting enzymes in the human body with extremely high catalytic efficiency. Mutations in the perlecan gene (HSPG2) have been identified in two classes of musculoskeletal conditions in the relatively mild Schwartz-Jampel syndrome and more severe but relatively rare neonatal lethal dyssegmental dysplasia, Silverman-Handmaker type (Arikawa-Hirasawa et al., 2001a; Arikawa-Hirasawa et al., 2001b; Arikawa-Hirasawa et al., 2002a). A unique mutation in perlecan in Schwartz-Jampel syndrome compromises the biomechanical competence of the intervertebral disc and is also associated with myotonia and various degrees of chondroplasia (Lin et al., 2021). We have also shown perlecan’s essential roles in the development of cartilaginous components with essential roles in endochondral ossification and development of the axial and appendicular skeleton. A greater understanding of the regulation of acetylcholinesterase in the NMJ is of relevance to research into how muscular dystrophy, muscular sclerosis and the erratic muscular movements of Parkinson’a disease occur (Jacobson et al., 2001; Aldunate et al., 2004). Changes in the basement membrane have also been observed in these and related medical conditions (Jayadev and Sherwood, 2017; Pozzi et al., 2017; Xu et al., 2018; Johnson, 2019; Punga et al., 2022). Perlecan is a functional component of basement membranes and its application in the repair of basal structures in the blood brain barrier following ischemic stroke continue to improve the recovery of this important structure (Bix, 2013; Bix et al., 2013). Synaptic HS-PGs have also now been identified with important roles in the regulation of neural responses. Incorrect assembly or distribution of these synaptic HS-PGs occurs in a number of neurological diseases showing their relevance to disease modification (Knight et al., 2011; Banerjee et al., 2017; Noborn and Sterky, 2021). Interest in the electro-conductive properties of GAGs in the design of dopant films in artificial synaptic membranes along with significant improvements in organic microelectronics and micro fabrication have led to improvement in functional artificial synapses capable of regulating neural responses (Wang et al., 2013; Gill et al., 2015; Buzanska et al., 2018). Artificial synapses that mimic biological synapse neuron-driven interactions have been proposed as next-generation memristor information systems driven by low energy requirements, high energy efficiency and fast computing speed (Liu et al., 2020; Zeng et al., 2021). These offer exciting possibilities in functional neural repair following trauma or disease. Basic studies on the biology of perlecan are thus warranted to optimally utilise recombinant perlecan domains for growth factor delivery and matrix stabilisation and in the regulation of cellular activity in many therapeutic procedures. Studies of this sort are likely to discover properties of perlecan of potential benefit to a diverse range of applications in repair biology. It is worthwhile noting that perlecan encodes significant biological information both in its core protein and attached HS side chains. A cursory examination of the HS interactome and advances in GAG analysis methodology amply demonstrate the biodiversity and interactive capability of HS interactions at the cell surface and their potential to control cell behaviour (Gómez Toledo et al., 2021; Szekeres et al., 2021; Vallet et al., 2021). Much still needs to be learned of the complex information conveyed by the GAG glycocode and how this information translates to the cellular mechanisms that drive tissue processes during development, growth, and disease. However, elucidation of the first draft of the GAG-protein interactome (Vallet et al., 2021) shows that this system has significant potential in the area of tissue repair processes and these useful properties are slowly becoming understood (Melrose, 2016; Hayes and Melrose, 2020). For example, perlecan domain I has been used as a FGF-2 and BMP-2 delivery vehicle in tissue repair strategies (Jha et al., 2009; Srinivasan et al., 2012). Recombinant perlecan domain V, a HS and CS substituted PG in its own right with equivalent stimulatory properties to full length perlecan (Lord and Whitelock, 2013), also shows promise in the repair of vascularized and cartilaginous tissues (Lord and Whitelock, 2013; Lord et al., 2014; Lin et al., 2020; Lord et al., 2020). Perlecan domains I and V thus offer exciting possibilities in repair biology that will continue to emerge and are eagerly anticipated.

## Glossary

AChE: Acetylcholinesterase
Ach: Acetylcholine
ACL: Anterior cruciate ligament
AD: Alzheimer’s disease
AFM: Atomic force microscopy
ApoE: Apolipoprotein E
BBB: Blood brain barrier
BM: Basement membrane
BMP: Bone morphogenetic protein
CCA: Common carotid artery
cGMP: Cyclic guanosine monophosphate
Col: Collagen
CS: Chondroitin sulphate
CTGF: Connective tissue growth factor
DDSH: Dyssegmental dysplasia Silverman-Handmaker type
DG: Dystroglycan
DMOAD: Disease modifying OA drug
Dok7: Docking protein 7
ECM: Extracellular matrix
EGF: Epidermal growth factor
ERK: Extracellular signal-regulated kinase
ET: Endothelin
ePTFE: Expanded polytetrafluoroethylene
FAK: Focal adhesion kinase
FGF: Fibroblast growth factor
FGFR: Fibroblast growth factor receptor
FRNK: Focal adhesion kinase–related non-kinase
GAG: Glycosaminoglycan
GM-CSF: Granulocyte macrophage-colony stimulating factor
G6b-B-R: Megakaryocyte-platelet receptor
HA: Hyaluronic acid
HEK: Human embryonic kidney
hESC: Human embryonic stem cell
HIF: Hypoxia-inducible factor
HGF: Hepatocyte growth factor
HS: Heparan Sulphate
HS-PG: Heparan Sulphate Proteoglycan
HSPG2: Heparan sulphate proteoglycan 2/perlecan
Ig: Immunoglobulin
IL: Interleukin
iPSC: Induced pluripotent stem cell
ITIM: Immunoreceptor tyrosine-based inhibition motif
IVD: Intervertebral disc
KO: Knockout
KS: Keratan sulphate
LDL: Low-density lipoprotein
LDLR: Low-density lipoprotein receptor
LG: Laminin type G domain
LRP4: Low-density lipoprotein receptor-related protein 4
Mab: Monoclonal antibody
MAPK: Mitogen-activated protein kinase
MCAO: Middle cerebral artery occlusion
MEK: Mitogen-activated protein kinase kinase
MEP: Motor end plate
MK: Megakaryocyte
MMP: Matrix metalloproteinase
MNB: Myelinated nerve bundle
MuSK: Muscle-Specific Kinase
NCAM: Neural cell adhesion molecule
NMJ: Neuromuscular junction
OA: Osteoarthritis
OGD: Oxygen-glucose deprivation
PCM: Pericellular matrix
PDGF: Platelet derived growth factor
PDGFRβ: Platelet derived growth factor receptor beta
PG: Proteoglycan
PRELP: Proline/arginine-rich end leucine-rich repeat protein
PTEN: Tumor suppressor phosphatase and tensin homolog
SAM: Synaptic adhesion/organizing molecules
SHP-2: Src homology region 2 domain-containing phosphatase-2
SJS: Schwartz Jampel Syndrome
SLRP: Small leucine rich proteoglycan
SMC: Smooth muscle cell
SMF: Striated muscle fibre
TGF-β: Transforming growth factor-β
TRP: Transient receptor potential
TUNEL: Terminal deoxynucleotidyl transferase
dUTP: nick end labeling
VEGF: Vascular endothelial growth factor
VEGF-R2: Vascular endothelial growth factor receptor 2
VLDL: Very low-density lipoprotein
WARP: Von Willebrand factor A‐domain‐related protein.
